# Parkinson's Disease Research on the African Continent: Obstacles and Opportunities

**DOI:** 10.3389/fneur.2020.00512

**Published:** 2020-06-19

**Authors:** Marieke C. J. Dekker, Toumany Coulibaly, Soraya Bardien, Owen A. Ross, Jonathan Carr, Morenikeji Komolafe

**Affiliations:** ^1^Department of Medicine and Pediatrics, Kilimanjaro Christian Medical Centre, Moshi, Tanzania; ^2^Service de Neurologie, Centre Hospitalier Universitaire du Point “G”, Bamako, Mali; ^3^Division of Molecular Biology and Human Genetics, Faculty of Medicine and Health Sciences, Stellenbosch University, Cape Town, South Africa; ^4^Department of Neuroscience, Mayo Clinic, Jacksonville, FL, United States; ^5^Department of Clinical Genomics, Mayo Clinic, Jacksonville, FL, United States; ^6^Division of Neurology, Faculty of Medicine and Health Sciences, Stellenbosch University, Cape Town, South Africa; ^7^Department of Medicine, College of Health Sciences, Obafemi Awolowo University, Ile-Ife, Nigeria

**Keywords:** Parkinson's disease, Africa, public health, awareness, epidemiology, genetics

## Abstract

The burden of Parkinson's disease (PD) is becoming increasingly important in the context of an aging African population. Although PD has been extensively investigated with respect to its environmental and genetic etiology in various populations across the globe, studies on the African continent remain limited. In this Perspective article, we review some of the obstacles that are limiting research and creating barriers for future studies. We summarize what research is being done in four sub-Saharan countries and what the key elements are that are needed to take research to the next level. We note that there is large variation in neurological and genetic research capacity across the continent, and many opportunities for unexplored areas in African PD research. Only a handful of countries possess appropriate infrastructure and personnel, whereas the majority have yet to develop such capacity. Resource-constrained environments strongly determines the possibilities of performing research locally, and unidirectional export of biological samples and genetic data remains a concern. Local-regional partnerships, in collaboration with global PD consortia, should form an ethically appropriate solution, which will lead to a reduction in inequality and promote capacity building on the African continent.

## Introduction

Mirroring global trends, life expectancy on the African continent has greatly increased in recent decades, paralleling economic growth, and related to a decline in a number of infectious diseases. The World Health Organization reports that overall life expectancy at birth in Africa is currently 61.2 years (1). As a result of improved control of the HIV epidemic, malaria and diarrhoeal diseases, non-communicable disorders (NCD) have become increasingly important as a public health concern for Africa (2), which is a global pattern observed initially in higher income countries. The common movement disorder, Parkinson's disease (PD), is one example of an important neurological NCD in the aging African population.

Exploring the epidemiology and genetic etiology of NCDs is essential in order to dissect out patterns of disease susceptibility, environmental clustering, and medication responses at a population level, as well as on an individual basis. In this Perspective article, we provide an overview of the main difficulties that we consider to be hindering the progress of PD epidemiological and genetic research on the African continent, and the solutions needed. We also provide summaries of the healthcare infrastructure in four African countries [represented by the four neurologists listed as authors; Tanzania (MD), Nigeria (MK), Mali (TC), and South Africa (JC)], and of the research done in these countries to illustrate the local obstacles but also the potential global opportunities for the field.

## Obstacles to PD Research on the African Continent

### Limited Number of Neurologists

Epidemiological patterns are heavily dependent on the power of detection of disease, which is an interplay of diagnostic factors as well as the accuracy of determining the correct diagnosis in the general population. In the case of PD, the first and foremost factor in this is the availability of neurologists. On the African continent, there is a wide discrepancy in the number of neurologists and medical facilities between different countries.

**Figure 1:**
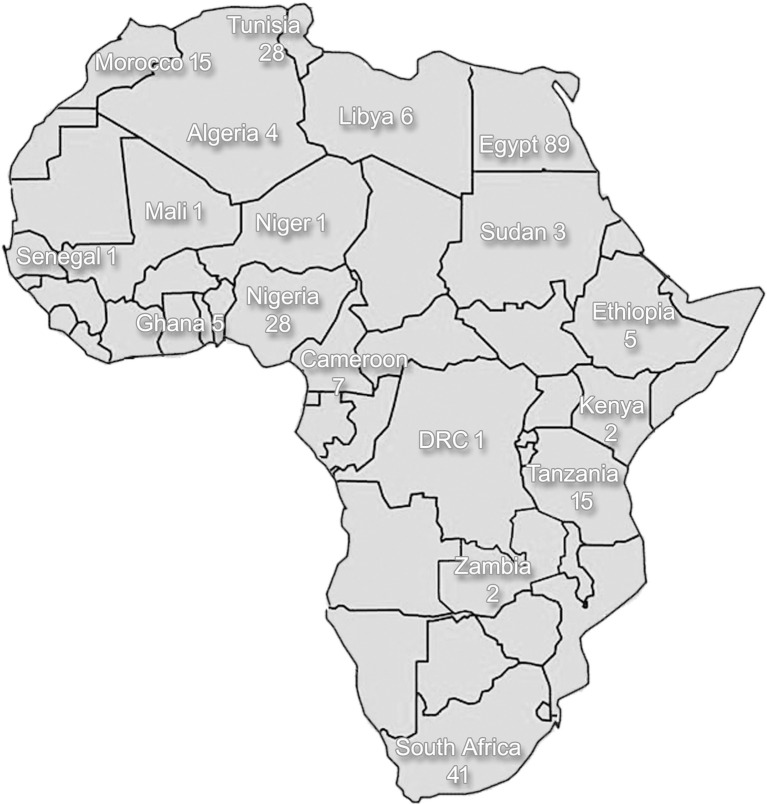
A schematic diagram of African countries indicating the number of published articles on Parkinson's disease per country (source: https://www.ncbi.nlm.nih.gov/pubmed/). The search was conducted in December 2019 and the term “Parkinson” was used in addition to the country name for all countries located on the African continent (Algeria-Zimbabwe); the ensuing results were then reviewed for being appropriate as a publication related to Parkinson's disease. Duration of search extended from 2019 until 1952.

Some urban centers may have comprehensive neurological and auxiliary services available, consisting of neurologists, neurosurgeons, neurophysiologists, and related equipment (such as electro-encephalography, electromyography, and nerve conduction studies). However, even in these centers, accessibility to such services by the general population is limited due to financial restrictions and barriers related to cultural perception of disease. Reviewing the situation in Africa as a whole, neurological services are either scarce or not available, as illustrated by a survey conducted in 2005 that included 11 countries that were entirely without neurological services (3). Currently, in most countries in Africa, there is still a dearth of neurologists, nurses, physiotherapists, and other allied professions due to limited training facilities for neurologists within Africa, as well as emigration of skilled personnel to more economically developed countries. Although, some research on the clinical and epidemiological aspect of PD has been conducted, genetics research of PD is limited in Africa, as a result of poor awareness and lack of facilities. The low number of scientific publications on PD mirrors the low density of neurological professionals (Figure 1). As can be seen in the figure, the Northern African Arabic countries bordering the Mediterranean Sea and South Africa at the tip of the continent are the two regions with better access to neurological surveillance and care than the remainder of Africa.

### Public Health Education and PD

A potential obstacle for access to neurological care may be preconceived beliefs that exist among the community about neurological conditions. Erroneous beliefs may arise from the absence of knowledge and education regarding a particular disorder, as well as culturally determined perceptions. Public health education is therefore of considerable importance. Absence of such education, or the cultural inappropriateness of educational content (for instance by direct translation of information leaflets and videos from other global regions) might lead not only to ongoing lack of recognition of medical disorders but also to missing out on the benefits of effective treatments. If one is not attuned to the specific culturally appropriate requirements of a region, the impression might arise that there is a resistance toward receiving educational information. However, with an approach adjusted to the specific ethnic, geographical, or religious needs of a target population, the same information may be better understood and therefore accepted.

### Stigma Associated With PD

In a Northern Tanzanian door-to-door survey in a semi urban setting, it appeared that many people suffering from PD met with various misconceptions about the disorder (4). Similarly, in a study conducted in South Africa, there was lack of knowledge about PD, with half of the members of the community believing that patients with PD should not live within the community (5). Ideas about guilt, witchcraft, and presumed mental disease all attribute to stigmatization. Such factors delay or prevent correct diagnosis or access to appropriate treatment for PD. The Tanzanian setting does have access to basic neurological services close to the survey area (6), which highlights the fact that targeting a community's perception of disease is potentially as important as is the improvement of structural facilities such as neurology clinics, laboratory diagnostics, and brain imaging. When educational material for patients and their caregivers is made available by a direct translation of quality material available from websites such as the International Parkinson and Movement Disorder Society (MDS; www.movementdisorders.org), it is expected to correctly reflect currently available evidence-based information. However, whether its contents will actually appeal to groups other than those in high-income regions, is less clear. In addition to appropriate translation, stigma due to superstitious beliefs and misconception (4, 5) also needs to be addressed in the educational material in order address target populations respectfully and effectively.

Consequently, we believe that some of the main challenges for PD research faced on the African continent are:

- Stigma of a visible impairment and the perception that the disease may be caused by a curse or is a bad omen.
- Delay in diagnosis and treatment due to traditional medicine being used as a first step for the majority of patients outside urbanized regions.
- Low rate of healthcare insurance coverage preventing affordability of long-term treatment in chronic disorders such as PD.
- Denial of a positive family history of a possibly genetic condition so as to prevent discredit to individuals or their relatives.

## PD Research in Four African Countries

In this section, we highlight the situation regarding PD research in four countries, Mali, Nigeria, South Africa, and Tanzania to illustrate the obstacles and the opportunities. A summary of the resources and infrastructure currently available for PD studies in each country is provided in Table 1. This table clearly shows the severe shortages of suitable resources, infrastructure and facilities in comparison to developed countries. However, despite this, high quality research has been done in these countries, as indicated below.

**Table 1:** Summary of healthcare and clinical resources available for the clinical management of Parkinson's disease in four sub-Saharan African countries.

	**Mali**	**Nigeria**	**South Africa**	**Tanzania**
Population size (millions)^*^	20	206	59	59
No. of neurologists^**^	20; (~1 per million people)	200; (~1 per million people)	Private sector 120; State sector 25; (~2.5 per million people)	8 (~1 per **7** million people)
No. of movement disorder specialists^**^	2	40 with special interest in movement disorders	Private sector, 15 with special interest in movement disorders; State sector, 2	None
Top three neurological conditions	Head trauma; stroke; CNS infections	Epilepsy; stroke; degenerative spinal cord disease	Stroke; epilepsy; peripheral neuropathy	Stroke; CNS infections; paraplegia
Prevalence of Parkinson's disease	Unknown	67/100,000 [community-based study; (13)]	Unknown	20/100,000 [community-based study; (7)]
Healthcare infrastructure	Three teaching hospitals; seven regional hospitals	85 tertiary hospitals (teaching hospitals and federal medical centres) of which 75 are public and 10 are private; 3,993 secondary hospitals of which ~75% are private	400 state hospitals; 200 private hospitals	269 hospitals of which 120 public or parastatal. Of these, seven teaching hospitals are connected to a medical school including four zonal referral hospitals; six additional specialized hospitals.
Medication funding	TB, HIV-AIDS, malaria treatment, and cesarean delivery free; Health insurance rate below 20%, most out of pocket	TB, HIV-AIDS treatment, and vaccination free; Health insurance rate below 10%, most out of pocket	Three payment options i.e., full paying, partially subsidized or free of cost (based on income); <10% of population has health insurance	TB, HIV-AIDS treatment and vaccinations free; Health insurance rate below 10%, most out of pocket
No. of CT/MRI scanners^**^	20 CT scanners; 4 MRI scanners	100 CT scanners; 50 MRI scanners	265 CT scanners; 150 MRI scanners	22 CT scanners; 8 MRI scanners
No. of PET/DAT scanners^**^	Unavailable	Unavailable	Six PET scanners; DAT unavailable	One PET scanner; DAT unavailable
Levodopa medication availability	Available only in the capital city and some districts. There is no insurance coverage for L-dopa medication	Subject to global availability, predominantly 25/250 strength; <5% covered by Health Insurance	Widespread availability, predominantly 25/100 and 25/250 strength	Subject to global availability, ~10% have coverage by healthcare insurance
DBS surgery availability	Unavailable	Unavailable	Available	Unavailable
No. of human geneticists	3	30–50	>200	Unavailable
No. of laboratories with human genetics expertise	Two labs [Neurosciences Department at the Point G Teaching Hospital, Faculty of Medicine, University of Sciences, Techniques and Technologies of Bamako (USTTB)]	30	>50	Two labs (Muhimbili National Hospital and Kilimanjaro Clinical Research Institute)

* *Country population size taken from https://www.worldometers.info/population/countries-in-africa-by-population/*.

** *Numbers are approximates as surveys have not been done*.

*DAT, Dopamine transporter; DBS, Deep brain stimulation; CNS, central nervous system; CT, Computerized tomography; MRI, magnetic resonance imaging; PET, positron emission tomography*.

Table 1 also shows the problems with treatment strategies for PD in Africa. In this setting, after clinically diagnosing parkinsonism, drug treatment usually starts with a levodopa/carbidopa trial. Dopamine agonists are unavailable in the majority of African countries. Treatment can be called unsuccessful when about one gram of levodopa/carbidopa daily for a number of weeks does not elicit a significant treatment response. Practically however, the high cost of the treatment may necessitate patients to terminate this titration prematurely, or to reduce dosage frequency to once daily or very low dosages. There will be a proportion of patients who would have responded better had there been no financial limitations. Physiotherapy is also a useful treatment modality, but is best given in limited sessions due to long travel distances and low resources. Physiotherapy in lower income regions is aimed at education and low frequency follow up: patients and relatives may attend for a few days consecutively, perform home exercises and return a number of months later.

### Tanzania

Tanzania, situated in East Africa, is one of the few countries in Sub-Saharan Africa where door-to-door prevalence data on PD are available from a survey of a semi urban and rural area (7). This survey also examined perception of disease, including that PD is considered to be an age-related phenomenon, which does not require treatment, or that it may be a punishment for having done something wrong (4, 7). The research group who conducted the survey has been funding levodopa therapy for newly diagnosed patients identified from the survey, in addition to following up the patients (8), and also studying physiotherapy interventions (9). A nearby tertiary referral center in Moshi, at the foot of Mount Kilimanjaro, also has neurologists available for this patient population (6). However, a limited number of patients follow up to obtain levodopa maintenance therapy, illustrating that there are additional, obstacles to care in an African rural population over and above the availability of a neurologist.

A levodopa containing crop, Mucuna Pruriens, is presently being studied for its medicinal properties in Moshi. Its use as monotherapy or add-on medication in PD has proven to be successful in Bolivia and Ghana (10, 11). The crop is being grown, and will be roasted and ground at the hospital premises using readily available facilities since Moshi is known for its coffee industry, which uses the same procedures. The availability of locally sourced medication of this nature may also allow patients with chronic illness to grow their own medicine at home, and titrate it themselves for daily use. In the framework of the above study, an assessment on candidate and pharmacologically relevant genes (e.g., Catechol-*O*-methyltransferase; *COMT*) will be performed. To date, only one genetically confirmed PD kindred is known from the East African region, which was identified in North Tanzania and is due to a homozygous *PRKN* deletion (12).

### Nigeria

Nigeria is the most populous and diverse nation in Africa with a growing population estimated to be over 200 million people, and home to many different ethnic groups speaking three major languages and over 250 other languages. Community based studies on the prevalence of PD obtained an age adjusted rate of 67 per 100,000 which is low compared to the frequency observed in African Americans (13). The clinical profile, etiology of Parkinsonism and PD and their complications have been described and are similar to the clinical profile in other regions of the world (14–16). Similarly, there have been studies on the non–motor features of neuropsychiatric impairment (17), cognitive impairment (18, 19), depression (20), gait instability (21), autonomic (22), gastrointestinal (23), and respiratory (24) involvement in PD. Studies to dissect the risk factors and etiology of PD among Nigerians include biochemical and pathological studies. Some authors observed the occurrence of Lewy bodies (25, 26), xenobiotics (27), and risk factors such as manganese among blacksmiths (28) and increased levels of trace metals (29). A few genetic studies have also been conducted and did not detect pathogenic mutations in *PRKN* (parkin), *LRRK*2, and *ATXN3* (30–32).

The challenges to care include low numbers of health care personnel, poor access to care, late presentation, as well as lack of medicine availability (33). However, new technologies, particularly telemedicine, have been identified as a promising area to improve access to care, especially for patients in rural communities (33). Educational campaigns and awareness efforts to tackle misconceptions as well as a multidisciplinary team care approach at the community level are anticipated to improve access and quality of care (34, 35).

### Mali

Mali is situated in the midst of the Sahara Desert between North African and sub-Saharan African countries. The demography is diverse and consists of Sub-Saharan ethnic groups living in the southern part of the country (black African origins) and nomadic racial groups (Arabic-Berber origins) living in the northern part of the country (36). The two ethnic groups share similar historic, cultural and religious traditions with each other, and there are high rates of consanguinity. These features are also shared with neighboring countries, namely the North African countries across the Sahara Desert and the sub-Saharan countries in the South. Almost all facilities and health care personnel in Mali are located in a geographic area representing <10% of the country, where only 14% of the population live (37). PD is not regarded as an urgent health priority when compared to the disease burden of infectious diseases and other NCDs. Long-term availability of medication, follow up and patient education are also lacking.

Due to a lack of trained movement disorders specialists and severe constraints in health care infrastructure, only two hospital-based studies of PD have been conducted in Mali. From January 2012 to November 2013, all cases of PD were collected using in-patient and out-patient visit data at Point G Hospital in Bamako, which is the main teaching hospital in Mali. Among the 8,372 patients seen at the Neurology Department, 60 patients (0.7%) had PD (38). Mostly, individuals aged 61–80 years were affected, the frequency of young onset cases was 12.2%, and a positive family history of PD was present in 7.3%. Another study done in 2016 revealed non-motor signs in 90% of all patients with PD (39). To date, there are no published genetic studies on PD patients from Mali.

### South Africa

South Africa has been described as a “melting pot,” since the country is ethnically diverse due to its history, comprising people from a range of different ancestral backgrounds. South Africa has reasonably well-established healthcare and facilities for clinical management of PD but there are wide discrepancies in facilities between different provinces (largely as a legacy of the apartheid era) and between the urban and rural areas. The PD research group is based in Cape Town and was initiated in 2006. As the country has some of the best resources and infrastructure for human genetics studies on the continent, the focus of the PD research group is to study the genetic etiology of the different ethnic groups by establishing a DNA bank of clinically well-characterized PD patients. Initially, the group concentrated on familial and early onset PD, of all ethnic origins, but more recently, a focus has been on recruitment of South African patients of Black African ancestry. The group has identified pathogenic mutations, albeit at low frequencies, in all of the commonly associated PD genes, as elaborated on in the next section. Recently, a *PTRHD1* mutation was identified in a Xhosa family with Parkinsonism and intellectual disability (40).

## Genetics Of PD in African Populations

As has been highlighted by many previous reports, genetic studies on African populations have been very limited (35, 41–43). All of the published studies and their findings are summarized in Supplementary Table 1. On the African continent, most of the work has been done on patients from North African Arabic countries where the frequency of the LRRK2 G2019S mutation was reported to be as high as 41% of patients (44) due to the presence of genetic founder effects. A number of studies have been conducted in South Africa but the mutation detection rate has been low (32, 40, 45–55). The other studies have been done in Nigeria (30–32), Tanzania (12), Zambia (56), and Ghana (57) but for the vast majority of the countries in Africa, no genetic studies have been reported. This is a striking omission since African populations have the oldest genomes and the greatest genetic diversity in the world, and are therefore likely to reveal novel insights into disease mechanisms and pathways underlying PD (58).

Notably, findings conducted on *LRRK2* and in particular, the G2019S mutation, have revealed interesting findings (Supplementary Table 1). Although common in North African Arabic populations, this mutation has not been identified in a single individual of Black African ancestry (30, 31, 49, 56, 57). A recent study conducted in South Africa found that 8 out of 647 patients screened were G2019S-carriers but all are of Ashkenazi Jewish origin except one (whose grandfather was German) (49). In a study on African Arabic patients in Tunisia, G2019S-carriers had similar PD symptoms to non-G2019S idiopathic PD cases but had a younger age at onset (AAO), a more benign phenotype and less cognitive impairment (59). In the South African study, the average AAO of the eight G2019S carriers was 56.6 years (SD 10.9), they had typical PD symptoms, and the homozygous mutation carrier did not exhibit a more severe disease to the others, although two patients had severe lower limb dystonia (49). It is plausible that patients of Black ancestry harbor other mutations in *LRRK2* but this would require comprehensive screening of all 51 exons of this gene.

In summary, genetic studies in African populations have the potential to be of great benefit for PD research globally but have largely been unexplored.

## Solutions Needed

In order to tackle the major challenges and obstacles to care of PD patients and to facilitate more research on this disorder, we believe the following issues need to be urgently addressed: lack of awareness and wrong perceptions, lack of trained personnel and the unavailability of drugs. To tackle the lack of awareness, awareness campaigns, and culture specific educational materials need to be developed in the local languages. Governments should improve awareness and reduce stigma through the use of radio and television jingles, adverts, and drama. Celebrities in each country who have the disease could be encouraged to talk about PD. This will improve awareness in the populations and may encourage patients to seek care earlier than they do currently. Observance of World Parkinson's Day (on 11 April annually) in healthcare institutions as well as obtaining sponsorship for other events such as quiz competitions and arts and cultural activities amongst school learners would be important. It has been observed that school learners can help to raise awareness of neurological disorders among the older members of the family (60).

In addition, in the short term, training of multidisciplinary teams comprising primary care physicians, and geriatricians as well as training of neurology nurses has been established in some parts of Africa and should be encouraged. The training of other team members such physiotherapists, occupational and speech therapists, and dieticians, should be promoted through local neurological and international societies such as the International Parkinson and Movement Disorder Society. A previous review suggested that tele-neurology can be deployed for training of health care workers through local, regional, and intercontinental networks (61).

To tackle the problem of the non-availability and un-affordability of drugs, a multisectorial strategy involving governments, pharmaceutical organizations, and other key stakeholders is necessary. It will be important for governments across Africa to include drugs for PD in the National Drug Formulary and to enroll patients in the health insurance programme. Incorporating PD care into health insurance systems will also enable patients to have access to neuroimaging (62). Neuroimaging facilities are becoming more widely available in Africa, but the cost of investigation is not affordable for most patients.

Finally, a holistic approach to care could be developed and implemented. The organization of PD support groups and clubs as well as organization of community-based rehabilitation will help in the care of patients living in rural communities.

## Conclusions

The genetic and environmental diversity across the African continent provides a wealth of information and opportunities for research into the epidemiological patterns of PD occurrence, its clinical phenotypes and the genetic and environmental causal factors. However, there is large variation in neurological and genetic research capacity across the African continent, and many unexplored areas in African PD research. Some countries are relatively well-equipped, but most are severely resource-constrained. A low resource environment strongly limits the possibilities of performing research locally, and therefore unidirectional export of genetic material to scientifically more developed countries remains a major concern. Local and regional partnerships can form an ethically appropriate solution, reducing inequalities, and promoting capacity building. There is also the possibility for collaboration of these partnerships with global consortia studying the genetic etiology of PD [Genetic Epidemiology of Parkinson's Disease (GEoPD; www.geopd.net) and The International Parkinson Disease Genomics Consortium (IPDGC; www.pdgenetics.org)], to provide training in genomics and bioinformatics to African scientists.

Furthermore, a major concern for the adequate treatment of PD patients is the availability of affordable levodopa-containing medication. Various African countries have difficulties in obtaining levodopa for their patients largely due to manufacturing capacity and supply chain constraints, which has prompted the need for development of alternative therapies. The levodopa-containing plant Mucuna Pruriens thrives in the subtropics and can be grown by patients for their own use. Options such as these and others should be considered to provide African-based solutions to uniquely African problems when dealing with the emerging PD pandemic (63).

## Author Contributions

MD contributed to design, conception, writing of the first draft, and editing of the manuscript. TC contributed to writing sections of the manuscript. SB and OR contributed to conception and editing. JC contributed to editing and compiled the figure. MK contributed to writing sections and editing. All authors reviewed and approved the final version of the manuscript.

## Conflict of Interest

The authors declare that the research was conducted in the absence of any commercial or financial relationships that could be construed as a potential conflict of interest. The handling editor declared a past co-authorship with one of the authors OR.
