# Supplementary material for: Parkinson's Disease Research on the African Continent: Obstacles and Opportunities
Source: Front Neurol. 2020 Jun 19;11:512. doi: 10.3389/fneur.2020.00512 (PMC7317302; doi:10.3389/fneur.2020.00512)
Supplement: Supplementary file 1 [file Table_1.docx]

**Supplementary Table S1**. Published mutation screening studies in African patients with Parkinson’s disease

| **Country** | **Patients and controls** | **Genes screened and method used** | **Main findings** | **Phenotype of mutation carriers** | **Reference** |
| --- | --- | --- | --- | --- | --- |
| **North African Arabic countries** | |  |  |  |  |
| **Algeria** | 1 multiplex family | PRKN (all 12 exons)  PCR and gel electrophoresis | PRKN homozygous deletion of exons 8 and 9 found in 3 affected siblings | Young AAO (< 45 years),  good response to levodopa | Lücking et al., 1998 |
|  | 106 patients | LRRK2 G2019S (exon 41)  Sanger sequencing | G2019S found in 34 patients (32%) | G2019S carriers had a similar age at onset to that of non-carriers. Behavioural abnormalities, mostly depression and hallucinations, and sleep disorders were more frequent in G2019S carriers | Belarbi et al., 2010 |
|  | 236 probands | LRRK2 G2019S (exon 41)  Sanger sequencing | G2019S found in 84 patients (36%); (77 heterozygotes and 7 homozygotes) | Mean age at onset in probands was 48.9 ± 12.1 | Troiano et al., 2010 |
|  |  |  |  |  |  |
| **Egypt** | 5 patients;  50 controls | GIGYF2 Asn56Ser and Asn457Thr;  TaqMan assay | Asn457Thr found in one patient, heterozygous;  Not present in controls | AAO 46 years presented with marked rigidity and bradykinesia and slight resting tremor, good response to levodopa | Zimprich et al., 2009 |
|  | 113 patients (sporadic PD)  87 controls | LRRK2 G2019S  PCR-RFLP/ TaqMan assay | G2019S found in 11 patients (9.7%); all heterozygous;  Not present in controls | Mean AAO was 55.3 ± 5.4 years, similar to non-carriers.  G2019S was associated with a higher degree of motor effect but does not seem to affect mentation or behavioural aspects of the disease | Hashad et al., 2011 |
|  |  |  |  |  |  |
|  |  |  |  |  |  |
| **Morocco** | 1 proband;  96 controls | Genome-wide copy number variation  (chromosomal microarray analysis);  LRRK2 (exon 41);  Sanger sequencing  PINK1 (all 8 exons)  Sanger sequencing | PINK1 homozygous L539F mutation found in one affected patient;  Not present in controls | AAO 54 years; patient presented with right akinetic-rigid syndrome with slight tremor in the hand. He also had neck pain and rapid eye movement behaviour disorder. Rapid progression and early cognitive impairment | Ben El Haj et al., 2016 |
|  | 1 multiplex family | WES | PINK1 homozygous A217D mutation found in two affected siblings | Early-onset disease (AAO 27 and 29 years), relatively mild and slowly progressive PD, early lower-limb symptoms and gait disturbance with asymmetrical onset | Norman et al., 2017 |
|  |  |  |  |  |  |
| **Tunisia** | 1 multiplex family | PRKN (all 12 exons)  Sanger sequencing | PRKN homozygous two-base AG deletion in exon 2 found in 3 affected siblings | AAO 21, 24, 34 years, clinical features included rest tremor, bradykinesia and rigidity, levopoda responsive | Gouider-Khouja et al. 2003 |
|  | 91 families | LRRK2 G2019S  Modified single base chain extension assay | G2019S found in 38 patients (42%); 23 heterozygous and 15 homozygous | G2019S carriers had an older AAO but had typical clinical features of PD | Ishihara et al. 2007 |
|  | 91 families (200 PD patients) | LRRK2 G2019S  Method not specified | G2019S found in 38 patients (42%) | Mean age at onset for affected individuals was 64±13 years for individuals with G2019S homozygous mutations, and was 68±12 years for individuals with heterozygous mutations, which was similar to individuals without G2019S mutations. | Warren et al., 2008 |
|  | 92 families (76 multiplex families comprised of 208 patients and 16 singleton families) | PINK1 (all 8 exons);  Sanger sequencing and exon dosage  LRRK2 (all 51 exons)  Saner sequencing | Four PINK1 mutations found in 14 families (Q129X, Q129fsX157, G440E, and Q456X); all homozygous | Younger AAO (36 ± 12 years) and longer disease duration, patients more likely to develop akinetic-rigid parkinsonism and had a slow progression | Ishihara-Paul et al., 2008 |
|  | 238 patients (sporadic PD)  371 controls | LRRK2 G2019  Taqman assay | G2019S found in 72 patients (30%); 60 heterozygous and 12 homozygous;  G2019S found in 7 controls (2%) | Clinical features and mean AAO (58± 10.7) of G2019S carriers were indistinguishable from noncarriers, and the distribution of akinetic-rigid, mixed, and tremor-dominant phenotypes was similar between the two groups | Hulihan et al., 2008 |
|  | 89 families (76 multiplex families comprised of 208 patients and 13 singleton families) | ATP13A2  Sanger sequencing (all 29 exons) | No mutations detected |  | Vilariño-Güell et al., 2009 |
|  | 33 probands | GBA (all 11 exons);  Sanger sequencing  GBA N370S screened in 395 patients and 372 controls by Taqman Assay | N370S found in one patient and 3 controls | No details provided | Nishioka et al., 2010 Neurosci Lett |
|  | 231 patients from 90 families | LRRK2 G2019S (exon 41)  TaqMan assay  PINK1 (all 8 exons)  Sanger sequencing and exon dosage  PRKN (all 12 exons)  Sanger sequencing and exon dosage | LRRK2: 73 G2019S carriers (20 homozygous)  PINK1: 42 mutation carriers  PRKN: 9 mutation carriers | AAO and mean disease onset in LRRK2 carriers (60 years) were similar to idiopathic PD but they had a more severe motor phenotype,  PINK1 carriers had younger mean AAO (35 years) and longer disease duration | Nishioka et al., 2010 J Neurol Neurosurg |
|  | 250 patients;  218 controls | LRRK2 G2019S;  KASP assay | G2019S found in 85 patients (33.6%); 76 heterozygous and 8 homozygous;  G2019S found in 3 controls  (1.3%); all heterozygous | The mean AAO was 64.1 ± 11.5 years; patients had typical PD | Landoulsi et al. 2017 |
|  | 1 multiplex family | 22 candidate genes;  next-generation sequencing gene panel | SYNJ1 heterozygous Leu1406Phefs*42 and Lys1321Glu found in two affected siblings | AAO at 16 and 21 years; one patient had generalized tonic-clonic seizures, moderate cognitive impairment, good response to levodopa | Ben Romdhan et al., 2018 |
|  |  |  |  |  |  |
| **Sub-Saharan African countries** | |  |  |  |  |
| **Ghana** | 53 probands + 1 affected sibling;  46 controls | LRRK2 (exons 31 and 41);  Sanger sequencing | No mutations detected |  | Cilia et al., 2012  (57) |
|  |  |  |  |  |  |
| **Nigeria** | 57 probands;  51 controls | LRRK2 (exons 31 and 41);  PRKN (all 12 exons);  ATXN3 (triplet repeat expansions);  Sanger sequencing | No mutations detected |  | Okubadejo et al., 2008 (31) |
|  | 126 patients;  54 controls | LRRK2 G2019S;  KASP assay | No mutations detected |  | Okubadejo et al., 2018 (30) |
|  | 14 probands | 751 candidate genes;  next-generation sequencing gene panel | No mutations detected (but candidate variants identified) |  | Oluwole et al., 2020 (32) |
|  |  |  |  |  |  |
| **South Africa** | 91 probands | PRKN (all 12 exons);  SSCP or HRM | Two patients had PRKN mutations:  ^≠^ PKRN homozygous exon 4 deletion (in two affected siblings);  ^*^ PKRN homozygous exon 3 and exon 4 deletion (one patient) | ^≠^ PRKN^:^ Early-onset (both AAO 27 years) with prominent dystonia  ^*^ PRKN: Early-onset (AAO 27) with foot dystonia and mild symptoms | Bardien et al., 2009 (45) |
|  | 88 probands | Copy number variations in known genes (MLPA kits P051 and P052); kits contain probes for LRRK2 G2019S and SNCA A30P | Four patients had mutations:  SNCA triplication (in one patient);  ^α^ PINK1 homozygous Y258X (in three affected siblings);  ^≠^ PKRN homozygous exon 4 deletion (in two affected siblings);  ^*^ PKRN homozygous exon 3 and exon 4 deletion (in one patient) | SNCA: Early-onset (AAO 46) with dementia, psychosis and autonomic failure  ^α^ PINK1: Early-onset (AAO 46, 45 and 47) with levodopa response | Keyser et al., 2010 Neurogenet (52) |
|  | 154 probands | PINK1 (all 8 exons);  HRM or Sanger sequencing | ^α^ PINK1 homozygous Y258X found in three affected siblings |  | Keyser et al. 2010 BBRC (51) |
|  | 205 probands | LRRK2 G2019S;  HRM | ^**^ G2019S found in 4 patients | Typical PD with AAO 42 -70 years, all with levodopa response | Bardien et al., 2010 (46) |
|  | 229 probands | PRKN (all 12 exons);  HRM,  Copy number variations in known genes (MLPA kits P051 and P052; kits contain probes for LRRK2 G2019S and SNCA A30P) | Seven patients had PRKN mutations:  PKRN heterozygous exon 2 duplication and heterozygous exon 9 deletion (one patient);  PRKN heterozygous P113fsX163 and heterozygous exon 3 deletion found in one patient;  PRKN heterozygous G430D and exon 4 deletion found in two unrelated patients;  PKRN heterozygous exons 2-6 duplication and heterozygous exon 5 duplication found in one patient;  ^≠^ PKRN homozygous exon 4 deletion found in two affected siblings;  ^*^ PKRN homozygous exon 3 and exon 4 deletion found in one patient | PRKN: Typical PD with AAO 25-56 years | Haylett et al. 2012 (50) |
|  | 418 probands | EIF4G1 R1205H and VPS35 D620N;  KASP assay | No mutations detected |  | Blanckenberg et al. 2014 (47) |
|  | 11 probands | Copy number variations in known genes (MLPA kit P051; kit contain probes for LRRK2 G2019S and SNCA A30P) | No mutations detected |  | Mahne et al., 2016 (53) |
|  | 1 multiplex family | Entire exome;  WES | PRKN two homozygous mutations ((R275W and M432V) found in 3 affected family members | Typical PD with AAO 48, 57 and 68 all with levodopa response, mild autonomic dysfunction | Carr et al. 2016 (48) |
|  | 210 probands | Copy number variations in known genes (MLPA kits P051 and P052; kit contain probes for LRRK2 G2019S and SNCA A30P) | No mutations detected |  | Van der Merwe et al. 2016 (55) |
|  | 1 multiplex family | Entire exome;  WES | PTRHD1 homozygous 28 bp deletion found in 3 affected family members | Intellectual disability with Parkinsonism (AAO 6, 26 and 30’s) with levodopa response, no autonomic dysfunction | Kuipers et al., 2018 (40) |
|  | 647 probands | LRRK2 G2019S;  HRM | G2019S found in 8 patients (1 homozygous and 7 heterozygous)  ^**^ four of these reported in Bardien et al. 2010 | Typical PD with AAO 42 – 70 years, symptoms were varied with patients presenting with impaired olfaction, REM sleep behaviour disorder, painful lower limb dystonia that was responsive to levodopa | Du Toit et al., 2019 (49) |
|  | 33 probands | 751 candidate genes;  next-generation sequencing gene panel | No mutations detected (but candidate variants identified) |  | Oluwole et al., 2020 (32) |
|  | 30 probands | GBA (all 11 exons);  Sanger sequencing | R120W (R159W; PD susceptibility allele) found in one patient | Typical PD with AAO 35 years | Mahungu et al., 2020 (54) |
|  |  |  |  |  |  |
| **Tanzania** | 1 multiplex family | Copy number variants in known PD genes;  MLPA kits P051 and P052 | PKRN homozygous exon 4 deletion found in two affected siblings | Early-onset (AAO 24 and 22 years) with levodopa response | Dekker et al., 2020 (12) |
|  |  |  |  |  |  |
| **Zambia** | 38 patients;  181controls | Subsets of patients were screened for:  LRRK2 (exons 29-48 or all 51 exons)  SNCA (all 8 exons)  PRKN (all 12 exons)  DJ-1 (all 8 exons);  Sanger sequencing;  Dosage analysis of LRRK2, SNCA, PRKN, PINK1 and DJ-1) | No mutations detected  (but heterozygous exon 2 and exon 4 deletions found in one patient) |  | Yonova-Doing et al., 2012 (56) |

AAO; age at onset of Parkinson’s disease; HRM, high-resolution melt; KASP, Kompetitive allele specific PCR; MLPA, Multiplex ligation-dependent probe amplification; SSCP; Single strand conformation polymorphism; WES, whole exome sequencing

≠, *, α, and ** symbols indicate that this is the same patient

**References** (ones that appear in the Supplementary Table but are not cited in the main article)

1. Lücking CB, Abbas N, Dürr A, Bonifati V, Bonnet AM, De Broucker T, De Michele G, Wood NW, Agid Y, Brice A. Homozygous deletions in parkin gene in European and North African families with autosomal recessive juvenile parkinsonism. *Lancet* (1998) **352**:1355–1356. doi:10.1016/S0140-6736(05)60746-5

2. Belarbi S, Hecham N, Lesage S, Kediha MI, Smail N, Benhassine T, Ysmail-Dahlouk F, Lohman E, Benhabyles B, Hamadouche T, et al. LRRK2 G2019S mutation in Parkinson’s disease: A neuropsychological and neuropsychiatric study in a large Algerian cohort. *Park Relat Disord* (2010) **16**:676–679. doi:10.1016/j.parkreldis.2010.09.003

3. Troiano AR, Elbaz A, Lohmann E, Belarbi S, Vidailhet M, Bonnet AM, Lesage S, Pollak P, Cazeneuve C, Borg M, et al. Low disease risk in relatives of North African LRRK2 Parkinson disease patients. *Neurology* (2010) **75**:1118–1119. doi:10.1212/WNL.0b013e3181f39a2e

4. Zimprich A, Schulte C, Reinthaler E, Haubenberger D, Balzar J, Lichtner P, El Tawil S, Edris S, Foki T, Pirker W, et al. PARK11 gene (GIGYF2) variants Asn56Ser and Asn457Thr are not pathogenic for Parkinson’s disease. *Park Relat Disord* (2009) **15**:532–534. doi:10.1016/j.parkreldis.2009.01.005

5. Hashad DI, Abou-Zeid AA, Achmawy GA, Allah HMOS, Saad MA. G2019S mutation of the leucine-rich repeat kinase 2 gene in a cohort of Egyptian patients with Parkinson’s disease. *Genet Test Mol Biomarkers* (2011) **15**:861–866. doi:10.1089/gtmb.2011.0016

6. Ben El Haj R, Regragui W, Tazi-Ahnini R, Skalli A, Bouslam N, Benomar A, Yahyaoui M, Bouhouche A. A novel homozygous p.L539F mutation identified in PINK1 gene in a moroccan patient with parkinsonism. *Biomed Res Int* (2016) **2016**:3460234. doi:10.1155/2016/3460234

7. Norman BP, Lubbe SJ, Tan M, Warren N, Morris HR. Early Onset Parkinson’s Disease in a family of Moroccan origin caused by a p.A217D mutation in PINK1: A case report. *BMC Neurol* (2017) **17**:153. doi:10.1186/s12883-017-0933-z

8. Gouider-Khouja N, Larnaout A, Amouri R, Sfar S, Belal S, Ben Hamida C, Ben Hamida M, Hattori N, Mizuno Y, Hentati F. Autosomal recessive parkinsonism linked to parkin gene in a Tunisian family. Clinical, genetic and pathological study. *Park Relat Disord* (2003) **9**:247–251. doi:10.1016/S1353-8020(03)00016-6

9. Ishihara L, Gibson RA, Warren L, Amouri R, Lyons K, Wielinski C, Hunter C, Swartz JE, Elango R, Akkari PA, et al. Screening for Lrrk2 G2019S and clinical comparison of Tunisian and North American Caucasian Parkinson’s disease families. *Mov Disord* (2007) **22**:55–61. doi:10.1002/mds.21180

10. Warren L, Gibson R, Ishihara L, Elango R, Xue Z, Akkari A, Ragone L, Pahwa R, Jankovic J, Nance M, et al. A founding LRRK2 haplotype shared by Tunisian, US, European and Middle Eastern families with Parkinson’s disease. *Park Relat Disord* (2008) **14**:77–80. doi:10.1016/j.parkreldis.2007.02.001

11. Ishihara-Paul L, Hulihan MM, Kachergus J, Upmanyu R, Warren L, Amouri R, Elango R, Prinjha RK, Soto A, Kefi M, et al. PINK1 mutations and parkinsonism. *Neurology* (2008) **71**:896–902. doi:10.1212/01.wnl.0000323812.40708.1f

12. Hulihan MM, Ishihara-Paul L, Kachergus J, Warren L, Amouri R, Elango R, Prinjha RK, Upmanyu R, Kefi M, Zouari M, et al. LRRK2 Gly2019Ser penetrance in Arab-Berber patients from Tunisia: a case-control genetic study. *Lancet Neurol* (2008) **7**:591–594. doi:10.1016/S1474-4422(08)70116-9

13. Vilariño-Güell C, Soto AI, Lincoln SJ, Yahmed S Ben, Kefi M, Heckman MG, Hulihan MM, Chai H, Diehl NN, Amouri R, et al. ATP13A2 variability in Parkinson disease. *Hum Mutat* (2009) **30**:406–410. doi:10.1002/humu.20877

14. Nishioka K, Vilariño-Güell C, Cobb SA, Kachergus JM, Ross OA, Wider C, Gibson RA, Hentati F, Farrer MJ. Glucocerebrosidase mutations are not a common risk factor for parkinson disease in North Africa. *Neurosci Lett* (2010) **477**:57–60. doi:10.1016/j.neulet.2009.11.066

15. Nishioka K, Kefi M, Jasinska-Myga B, Wider C, Vilariño-Güell C, Ross OA, Heckman MG, Middleton LT, Ishihara-Paul L, Gibson RA, et al. A comparative study of LRRK2, PINK1 and genetically undefined familial Parkinson’s disease. *J Neurol Neurosurg Psychiatry* (2010) **81**:391–395. doi:10.1136/jnnp.2009.185231

16. Landoulsi Z, Benromdhan S, Ben Djebara M, Damak M, Dallali H, Kefi R, Abdelhak S, Gargouri-Berrechid A, Mhiri C, Gouider R. Using KASP technique to screen LRRK2 G2019S mutation in a large Tunisian cohort. *BMC Med Genet* (2017) **18**:70. doi:10.1186/s12881-017-0432-5

17. Ben Romdhan S, Sakka S, Farhat N, Triki S, Dammak M, Mhiri C. A Novel SYNJ1 Mutation in a Tunisian Family with Juvenile Parkinson’s Disease Associated with Epilepsy. *J Mol Neurosci* (2018) **66**:273–278. doi:10.1007/s12031-018-1167-2
